# Membrane bound modified form of clade B Env, JRCSF is suitable for immunogen design as it is efficiently cleaved and displays all the broadly neutralizing epitopes including V2 and C2 domain-dependent conformational epitopes

**DOI:** 10.1186/s12977-016-0312-7

**Published:** 2016-11-21

**Authors:** Supratik Das, Saikat Boliar, Nivedita Mitra, Sweety Samal, Manish Bansal, Wayne C. Koff, Bimal K. Chakrabarti

**Affiliations:** 1 THSTI-IAVI HIV Vaccine Design Program, Translational Health Science and Technology Institute, NCR Biotech Science Cluster, 3rd Milestone, Faridabad-Gurgaon Expressway, PO box #04, Faridabad, Haryana 121001 India; 2 International AIDS Vaccine Initiative, New York, NY USA

**Keywords:** HIV, Envelope, Efficiently cleaved, Broadly neutralizing antibody binding, Immunogen design

## Abstract

**Background:**

Antigenicity of HIV-1 envelope proteins (Envs) of both lab-adapted and primary isolates expressed on the cell surface rarely match with in vitro neutralization of viruses, pseudo-typed with corresponding Envs. Often, both neutralizing and non-neutralizing antibodies bind to Envs expressed on the cell membrane. This could be due to the lack of efficient cleavage of Env expressed on the cell surface. Naturally occurring, efficiently cleaved Envs with appropriate antigenic properties are relatively rare. Given viral diversity it is essential to increase the pool of candidate Envs suitable for immunogen design. Previously, it has been reported that JRFL Env is the only clade B Env, which is efficiently cleaved on the cell surface and retains desirable antigenic properties. JRCSF is a clade B Env isolated from the same patient as JRFL. JRCSF Env has not been explored aggressively for designing immunogen as the binding characteristics of JRCSF Env to broadly neutralizing antibodies on the cell surface and its cleavage status are unknown.

**Results:**

Although JRCSF preferentially binds to most of the other gp120-directed neutralizing antibodies and cleavage dependent antibody, PGT151 efficiently, it binds poorly to CD4-binding-site-directed (CD4-bs-directed) neutralizing antibodies on cell surface. Membrane bound form of modified JRCSF Env containing the N197D mutation binds to CD4-bs-directed neutralizing antibodies better than JRFL, without debilitating its ability to bind quaternary epitope-directed neutralizing antibodies or exposing the CD4i antibody epitopes. In comparison to JRFL (E168K), JRCSF Env binds more efficiently to PG9/PGT145 class of V1/V2-directed conformational antibodies. Biochemical, cell surface staining and gp120 shedding experiments suggest that JRCSF is efficiently cleaved on the cell surface.

**Conclusions:**

Binding of JRCSF Env expressed on cell surface to the various HIV-1 Env-directed antibodies has not been reported earlier. Here, for the first time, we report that compared to JRFL, JRCSF displays epitopes for a larger number of broadly neutralizing antibodies and is also efficiently cleaved when expressed on the cell surface. Thus, considering the diversity of viral Envs and the discovery of conformation dependent glycan-directed antibodies in HIV-1 infected individuals, an innately cleaved JRCSF Env as present on the viral membrane and displaying those distinct epitopes may be an important candidate for immunogen design.

**Electronic supplementary material:**

The online version of this article (doi:10.1186/s12977-016-0312-7) contains supplementary material, which is available to authorized users.

## Background

In the past decade, one of the major strategies to develop a vaccine for HIV-1 has focused on activating the humoral immune response against genetically variable envelope glycoprotein (Env) to elicit broadly neutralizing antibodies (bNAbs) [1–4]. The Env mediates entry of the virion into target cells, undergoing conformational changes after engaging the primary receptor, CD4, and then binding to the co-receptor, usually CCR5; which ultimately leads to virus-to-cell membrane fusion and entry of virus into target cells [5–18]. Much has been learned from bNAbs binding and characterization studies on Env. For example, some of these bNAbs are dependent on the trimeric conformation of Env and binding of one such recently isolated bNAb, PGT151 is cleavage dependent [19, 20]. These studies provide insights to help guide immunogen design to elicit such bNAbs; the major challenge currently impeding HIV vaccine development.

The current paradigm is that an important starting point for immunogen design is that it should mimic the native Env as present on the virus, since the functional, trimeric Env spikes selectively resist binding by all but the most precisely targeted NAbs [21, 22]. The non-covalently linked trimeric form of the Envs are arranged on the membrane after the glycoprotein undergoes maturation wherein the gp160 precursor is proteolytically cleaved by cellular enzymes belonging to the furin family into the gp120 (SU) and gp41 (TM) subunits [13].

Several reports have demonstrated that the efficient cleavage of gp160 is a prerequisite for preferential binding to neutralizing (NAbs) over non-neutralizing antibodies (non-NAbs) on the cell surface [21, 23, 24]. Furthermore, the gp120-gp41 interaction is labile making it difficult to develop soluble form of Env proteins that are metastable but retains a trimeric, native-like conformation. This problem has been circumvented by generating SOSIP versions (containing A501C, T605C, I559P mutations in BG505) of the cleaved, C-terminus truncated Env glycoprotein to aa664 and purifying the trimeric form [25]. Purified BG505SOSIP.664 (MPER deleted and contains the T332N substitution) forms a cleaved, trimeric, native-like Env protein that binds preferentially to bNAbs but generally not to non-NAbs [21, 25]. Recently, similar versions of JRFL, B41 (clade B) and 16055 (clade C) have been generated, purified and demonstrated similar properties [22, 26]. This current vaccine approach generally elicits autologous tier 2 nAbs, but not bNAbs [27], indicating that additional modifications to the immunogen and/or immunization strategy will be needed for induction of bNAbs

The cleaved, native, trimeric form of Env immunogen can be realized through DNA vaccination as well. DNA prime-protein boost vaccination strategy, including HIV-1 Env, has also been used in several clinical trials e.g. RV144. Studies have also shown that DNA prime-soluble protein boost regimens frequently elicit qualitatively better NAbs compared to protein-only regimens [28–30]. This could be due to the expression of efficiently cleaved Env after DNA vaccination. However, naturally occurring, efficiently cleaved Envs that have been identified are rare. The clade B Env, JRFL was identified as the first naturally cleaved Env on the cell surface [24] and we have recently found a clade C Env 4-2.J41 from India that is also efficiently cleaved on the cell surface [23]. Using JRFL as a model system it has been demonstrated in non-human primates (NHPs) that a DNA prime followed by non-native trimeric Env boost elicits low-titer neutralization activity against some difficult to neutralize Tier-2 HIV-1 viruses [28]. In JRFL Env however, the epitopes for many of the recently discovered conformation-dependent glycan-directed antibodies are not present [3].

It appears from recent reports that Envs like JRFL have a glycan-deficient gap that could elicit antibody directed to a conserved site to which access is usually modulated by a glycan for virus pseudotyped with other Envs [31]. Furthermore, in one study, with JRCSF gp120 DNA prime, followed by gp120 protein boost, NAbs against some tier 2 viruses were induced [32]. Therefore, in our search for additional Envs, which may be used for genetic vaccination and in developing soluble immunogens, we studied JRCSF, isolated from the cerebrospinal fluid of the same patient from which the well-studied clade B Env, JRFL (isolated from the frontal lobe) was isolated. Though there is ample information about the antigenic properties in relation to the efficiency of cleavage of JRFL Env [24], sufficient studies have not yet been done with JRCSF. Moreover, data related to the differential neutralizing sensitivity between JRFL and JRCSF to CD4-bs-directed NAbs and other gp120-directed antibodies are in literature [3]. JRFL and JRCSF show 74 amino acid differences spread all over the gp160 polypeptide (Additional file 1: Figure 1).

Here, we show for the first time that JRCSF is an efficiently cleaved Env when expressed on cell surface and binds preferentially to most gp120-directed NAbs other than those directed against the CD4-bs. In addition, it does not bind to non-NAbs. The binding ability of JRCSF to CD4-bs-directed NAbs could be recovered without changing its native conformation through simple modification. JRCSF (N197D) appears to bind more efficiently to CD4-bs-directed NAbs than JRFL while JRCSF binds to PG9/PGT145 class of antibodies more efficiently than JRFL (E168K). As JRCSF Env is efficiently cleaved and can bind preferentially to all the different classes of quaternary epitope-dependent but glycan directed bNAbs when expressed on the cell surface, it may be better suited for use as a platform for designing immunogen due to its ability to bind to bNAbs targeting the trimer-specific V2 “cap” while retaining its ability to bind CD4-bs-directed bNAbs through the N197D mutation. The implications of these findings are discussed.

## Methods

### DNA constructs, cell lines and antibodies

Swapping mutants of JRCSF in pSVIII-env plasmid backbone were constructed by replacing the corresponding regions of JRFL by PCR amplification using *Pfu* polymerase following manufacturer’s protocol. Briefly, these PCR amplified fragments were gel purified and used as primers along with JRCSF as template and PCR amplified with *Phusion* polymerase according to the manufacturer’s protocol. The reaction mixtures were digested with *Dpn*1, transformed into competent cells and plated onto LB-ampicillin plates. The swapped JRCSF variants were confirmed by sequencing of the plasmid DNA isolated from unique colonies. For site-directed mutagenesis, mutagenic primers were designed according to the Quikchange site directed mutagenesis kit manual (Qiagen) and PCR was carried out with *Phusion* polymerase. The reaction mixtures were digested with *Dpn*1, transformed into competent cells and plated onto LB-ampicillin plates. The mutations were confirmed by sequencing of the plasmid DNA isolated from unique colonies. TZM-bl and 293T cells were obtained from NIH AIDS Reagent Program and ATCC, respectively. They were maintained in DMEM (Dulbecco’s modified Eagle medium) containing 10% HIFBS (heat inactivated fetal bovine serum), 20 mM l-glutamine, 100 U/ml penicillin, and 100 μg/ml streptomycin. Broadly neutralizing antibodies (VRC01, b12, PGT121, PGT145, PGT151, PG9, PG16, 10E8, 2G12) and non-neutralizing antibodies (F105, b6, 39F, and 17b) were obtained from the IAVI Neutralizing Antibody Center (NAC) at TSRI, La Jolla, California.

### FACS-based cell surface expression assay

FACS-based cell surface expression assay was carried out as described previously [33]. It is to be noted here that we have used the full-length clones of both JRFL and JRCSF in all our studies. Briefly, 293T cells were transiently transfected with pSVIII-Env plasmids expressing different forms of Env protein under the control of the HIV-1 LTR and pc-tat plasmid expressing Tat protein at the ratio of 20:1. 36–48 h post transfection, cells were harvested, washed three times with FACS buffer 1 (DMEM + 10% HIFBS) and stained with varying concentrations of monoclonal antibodies (neutralizing and non-neutralizing) for 1 h at room temperature (RT). The cells were washed three times with FACS buffer 1 and then stained with PE-conjugated goat anti-human secondary antibody (1:200 dilutions, Jackson ImmunoResearch) for 1 h at RT. The cells were again washed three times with FACS buffer 2 (PBS + 10% HIFBS) and fixed with 0.5% paraformaldehyde. The stained cells were then analyzed in a FACS Canto analyzer (BD Biosciences) and data analyzed with FlowJo software (version 10.0.6, Tree Star Inc).

### Plasma membrane fraction isolation, immunoprecipitation, gp120 shedding assays

Plasma membrane fractions of 293T cells transfected with different Envs were isolated using the Plasma Membrane Protein Isolation kit (Abcam) following the manufacturer’s protocol. PM fractions were resuspended in lysis buffer (10 mM Tris–HCl (pH 8.0), 150 mM NaCl, 1% Triton-X, 1 mM DTT and protease inhibitors) and immunoprecipitated (with rotation) O/N at 4 °C with bNAbs. Next day the mixture was incubated (with rotation) for 1 h at 4 °C with Immobilized Protein G resins (G Biosciences) and washed three times with PBS + 1% Triton-X. Washed beads were analyzed by western blot analysis using HIVIG Ab.

CD4-induced shedding of gp120 was assayed as described previously [23]. Briefly, cells transfected with plasmids expressing JRCSF Env were harvested and washed with FACS buffer 2 and then incubated with or without 50 µg/ml sCD4-183 (NIH AIDS Reagent) for 1 h at 4 °C with intermittent mixing. Cells were centrifuged and the supernatant subjected to ELISA as described previously [23] except that mAb, 39F was used as the primary antibody (1:1000 dilution) and peroxidase-coupled goat anti-human IgG (Jackson ImmunoResearch, Cat No. 109-036-008) was used as secondary antibody.

## Results

### Differential binding to CD4-bs bNAbs by JRFL and JRCSF

**Fig. 1:**
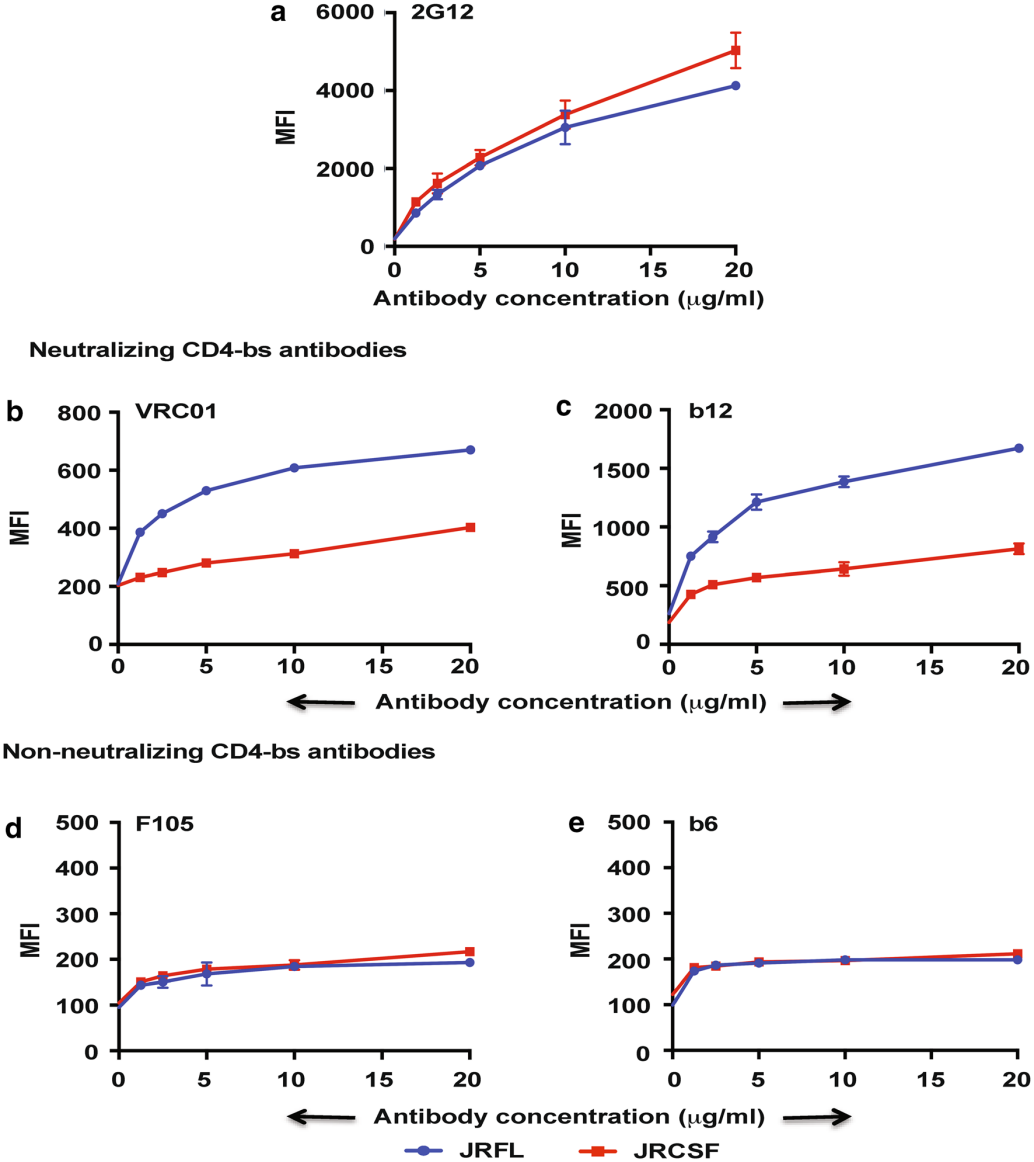
Differential binding of JRFL and JRCSF to CD4-bs-directed bNAbs b12 and VRC01. FACS-based cell surface staining assay was used to measure binding of **a** glycan-dependent antibody 2G12, **b**, **c** CD4-bs-directed bNAbs b12 and VRC01, and **d**, **e** CD4-bs-directed non-NAbs F105 and b6 to JRFL and JRCSF over a range of antibody concentration

Efficient binding to neutralizing antibodies and weak or no binding to non-neutralizing antibodies by Env on the cell surface can be used as a marker for determining the efficiency of cleavage of the Env gp160 to its constituent subunits [3, 23, 24]. It has been previously demonstrated that pseudoviruses prepared with either JRFL or JRCSF Env are neutralized by CD4-bs neutralizing antibodies [3, 34]. However, JRFL pseudotyped viruses are neutralized at least fivefold to tenfold more efficiently than JRCSF pseudotyped viruses by CD4-bs bNAbs (Table 1). In cell surface FACS-based staining assays, Walker et al. [3] had observed a differential binding of JRCSF and JRFL with the CD4-bs bNAb b12. In order to characterize JRCSF, we first determined its ability to bind to CD4-bs bNAbs b12 and VRC01 and the CD4-bs non-neutralizing Abs F105 and b6 by FACS-based cell surface staining assays (Fig. 1). We used binding to 2G12 as a marker for determining levels of expression. We observed that while JRFL binds efficiently to b12 and VRC01, JRCSF, in contrast, binds only marginally to CD4-bs bNAbs on the cell surface (Fig. 1b, c). Both Envs bind poorly to the non-NAbs F105 and b6 (Fig. 1d, e) while they bind to a similar extent to 2G12 suggesting equivalent levels of expression (Fig. 1a). Taken together these studies suggest that JRCSF is defective in its ability to bind CD4-bs bNAbs as compared to JRFL. However, like JRFL, JRCSF binds CD4-bs-directed non-NAbs poorly.

**Table 1:** Representative neutralization data of JRFL and JRCSF pseudotyped viruses [3, 34, 38]

mAb epitope	mAb	JRFL	JRCSF
CD4-bs	b12	+++	++
	VRC01	+++	++
	NIH45-46	++++	+++
	CH103	++++	+++
	3BNC117	++++	+++
	8ANC131	+++	++
CD4i	17b	−	−
V1V2V3 glycan-dependent	PG9	−	++++
	PG16	−	++++
	PGT145	+	++++
	PGT121	+++	+++
	PGT128	++++	++++
	2G12	+	++
MPER	10E8	++	++
	4E10	+	+
	2F5	+	+
FP	VRC34	+	±

+ IC_50_: 1–10

++ IC_50_: 0.1–1

+++ IC_50_: 0.01–0.1

++++ IC_50_: <0.01

− IC_50_ > 50

### JRCSF binds preferentially to neutralizing antibodies

**Fig. 2:**
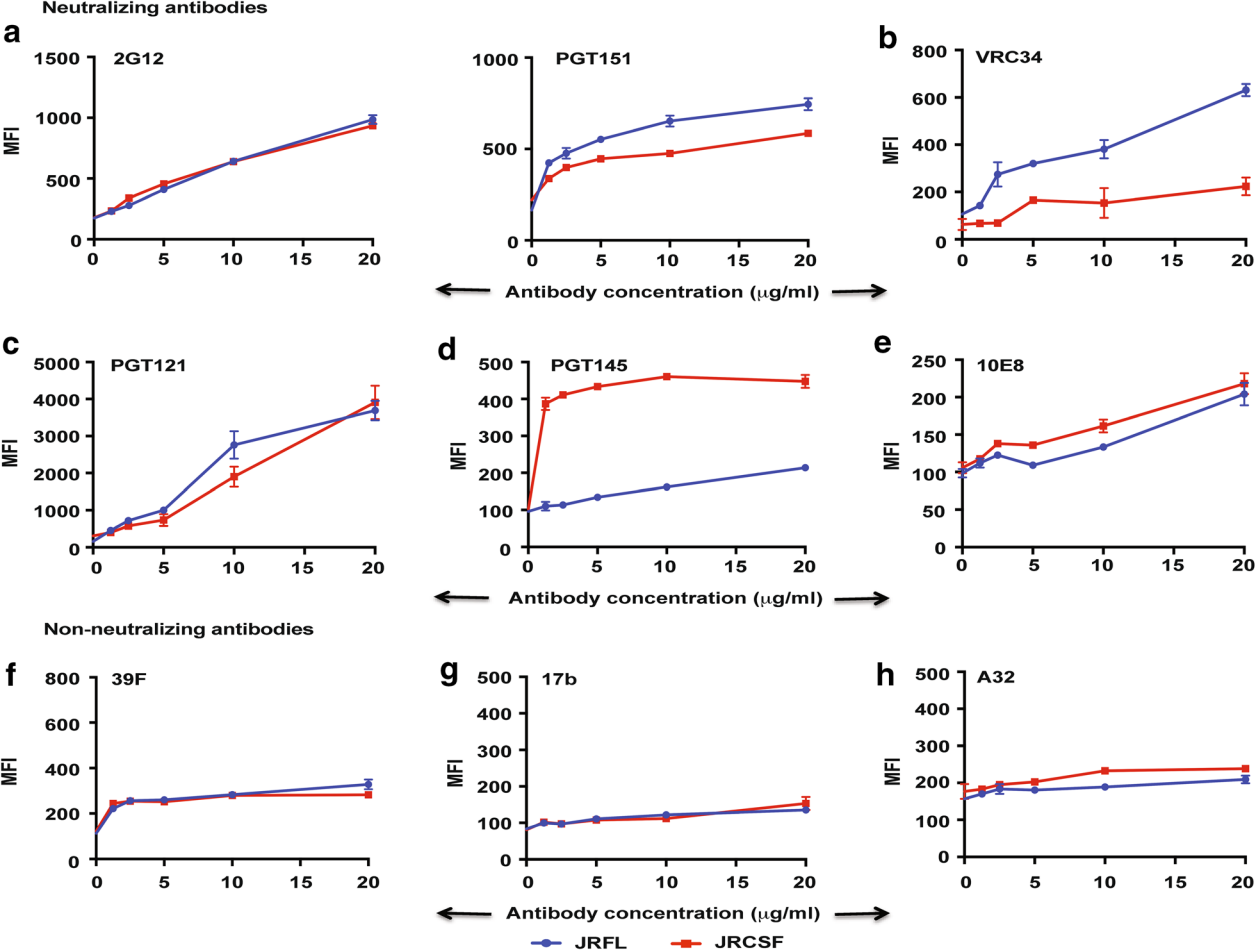
FACS-based cell surface staining assay of JRFL and JRCSF with **a**–**e** the neutralizing Abs 2G12, PGT151, VRC34, PGT121, PGT145 and 10E8 and **f**–**h** non-neutralizing Abs, 39F, 17b and A32 over a range of antibody concentrations

It has been reported previously that naturally cleaved HIV-1 Envs, JRFL and 4-2.J41 when expressed on the cell surface, selectively binds to neutralizing antibodies [23, 24, 33]. Uncleaved Envs can bind to both neutralizing and non-neutralizing antibodies [21, 23, 24, 33, 35]. The soluble, native-like trimers of BG505SOSIP.664 [25, 35, 36] and some other soluble native trimeric proteins have been made following this principle [22, 37]. In order to investigate whether JRCSF demonstrates similar properties, we wanted to check the binding ability of JRCSF Env expressed on the cell surface to both neutralizing and non-neutralizing antibodies. To compare the binding ability of both bNAbs targeting diverse epitopes (via the trimer selective V2 “cap”, the V3 region and the MPER region) and non-neutralizing antibodies to JRFL and JRCSF Envs, a FACS-based cell surface antibody binding assay was carried out with different neutralizing and non-neutralizing antibodies over a range of antibody concentrations as indicated in Fig. 2. We observed that both JRFL and JRCSF bind to the glycan-dependent bNAb 2G12 with equal efficiency (Fig. 2a) suggesting that they are expressed at similar levels. They also bind to the cleavage-specific and trimer-dependent bNAb PGT151 with equal efficiency (Fig. 2a). In addition, JRFL and JRCSF bind to V3 dependent conformational antibody, PGT121 and MPER targeted antibody, 10E8 to similar extent (Fig. 2c, e). As determined previously for JRFL with 2F5 and 4E10 [33], binding to these MPER-directed antibodies was relatively less efficient with JRCSF. On the other hand, in agreement with previous studies [3], while JRCSF binds efficiently to the V2 region targeted, glycan and quaternary structure-dependent antibodies PG9, PG16 (data not shown) and PGT145 (Fig. 2d), JRFL does not bind to these antibodies. Binding to this class of bNabs can be restored in JRFL by the E168K mutation [22]. Furthermore, while JRFL binds to the newly discovered fusion peptide-directed bNAb, VRC34 efficiently, JRCSF binds to it only marginally on cell surface (Fig. 2b). In pseudovirus neutralization assay, VRC34 modestly neutralizes JRFL pseudotyped viruses with an IC_50_ value of 1.1 µg/ml, while the IC_50_ for JRCSF pseudotyped viruses is >10 µg/ml (Table 1) suggesting that in JRCSF, the epitope for VRC34 is either inaccessible or is not in the same conformation as in JRFL. We also compared the ability of JRFL and JRCSF to bind to non-neutralizing antibodies. JRFL and JRCSF bind only marginally and to a similar extent to the non-neutralizing antibodies, 39F, A32 and the CD4i antibody 17b (Fig. 2f–h). Thus, both JRFL and JRCSF bind preferentially to neutralizing antibodies targeting the gp120-gp41 interface, V3 region as well as MPER epitopes. In contrast to JRFL, JRCSF binds to the quaternary structure-dependent V2 epitope-targeted antibodies PG9, PG16 and PGT145 efficiently. Both Envs bind to non-neutralizing antibodies poorly. These findings with JRFL and JRCSF expressed on the cell surface are in agreement with pseudovirus neutralization data (Table 1) performed in our laboratory (in this manuscript and data not shown) and also reported previously [3, 34, 38].

### JRCSF binding to PG9/PGT145 class of antibodies is dependent on entire V2 domain

**Fig. 3:**
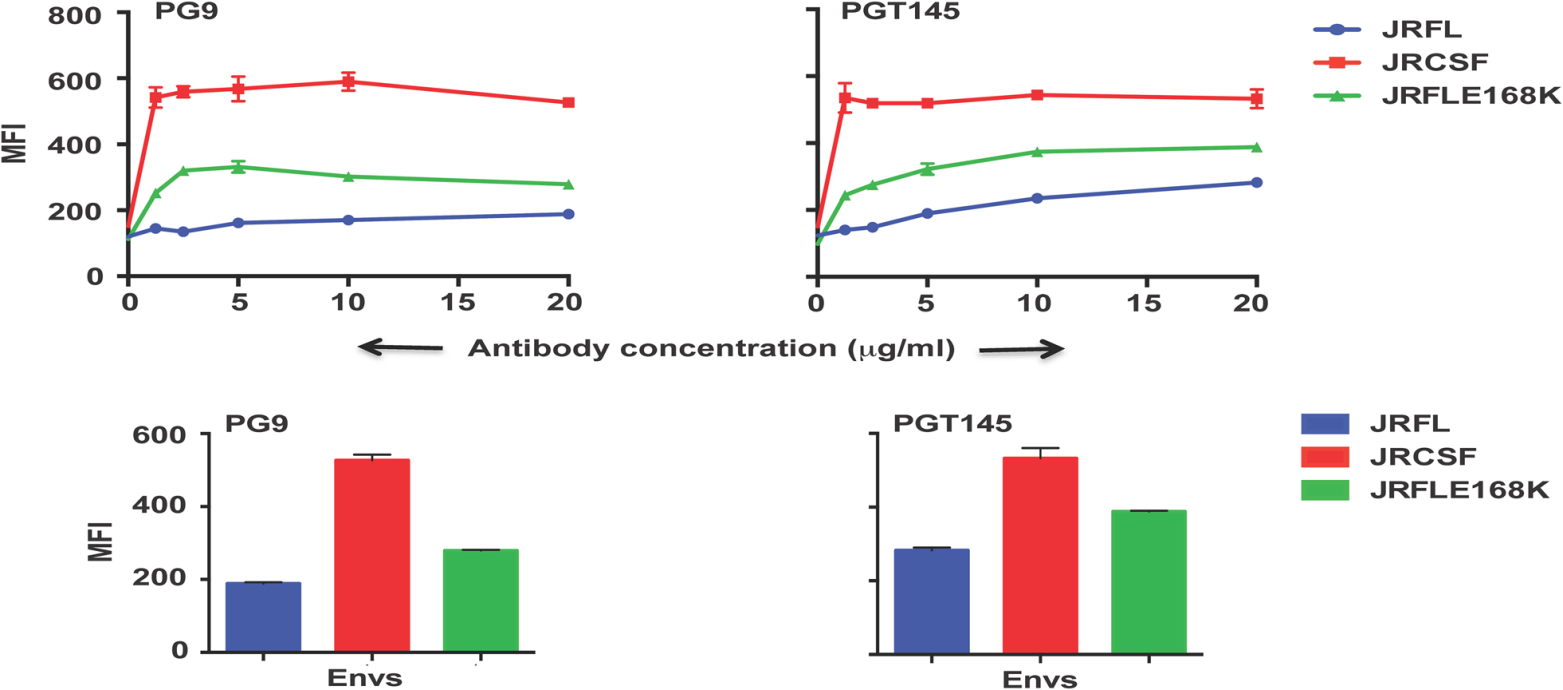
Comparative binding of JRFL, JRCSF and JRFL (E168K) to V2 “cap” targeted glycan and conformation-dependent Abs PG9 and PGT145 over a range of antibody concentrations by FACS-based cell surface staining assay. The bar diagram represents the binding of the respective antibodies to wild type and mutant Envs at 20 µg/ml of antibody concentration

It has been previously demonstrated that JRFL, in contrast to JRCSF, binds weakly to PG9 and PG16 antibodies when expressed on the cell surface [3]. In this report, we have confirmed that JRCSF binds potently to PG9 and PG16 while JRFL binds poorly to those antibodies. We have also shown that JRCSF binds strongly to PGT145 but not JRFL suggesting that JRCSF, in contrast to JRFL, has the advantage of the ability to bind to this class of bNAbs. Furthermore, the report by Walker et al. [3] has suggested that the conformational antibodies directed to the V2 and V3 variable loops and to a lesser extent co-receptor directed antibodies compete with PG9 for gp120 binding in ELISA and amino acid residues located in the conserved regions of the V2 and V3 loops play an important role in antibody mediated neutralization assays of pseudotyped viruses. Asparagines, N156 and N160 were particularly critical for recognition by PG9 and PG16 antibodies [3]. Besides JRFL, which is resistant to PG9 and PG16, was rendered sensitive to these antibodies by an E168K mutation in the V2 loop [3]. So we compared JRFL (E168K) with JRCSF for their ability to bind PG9 and PGT145 in FACS based cell surface binding assay (Fig. 3). Although JRFL (E168K) showed increased binding to PG9 and PGT145 as compared to JRFL it was not as efficient as JRCSF (Fig. 3, left and right panel) in its binding capability suggesting that JRCSF is a better Env than JRFL (E168K) with regards to its ability to efficiently bind this class of bNAbs.

**Fig. 4:**
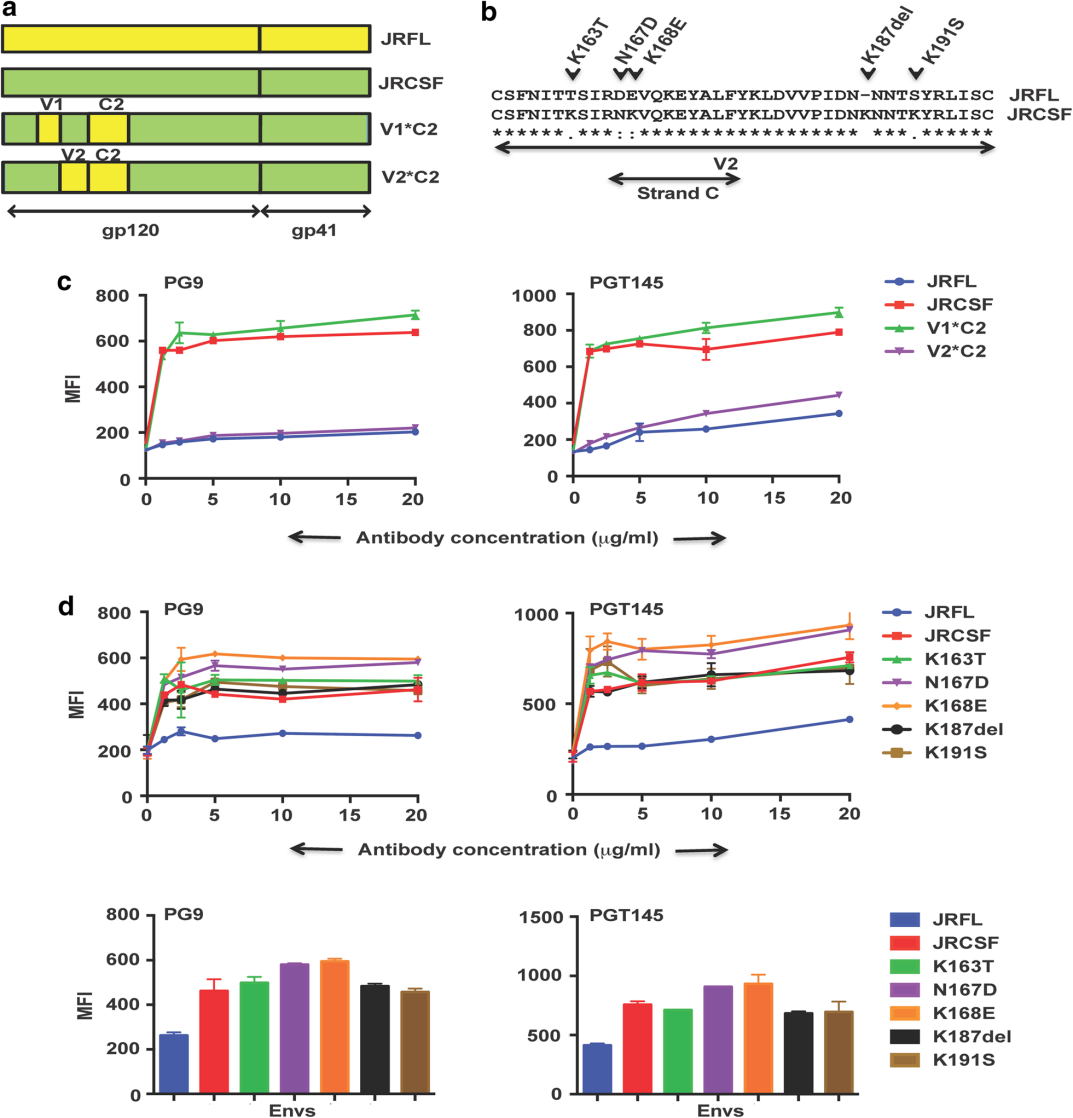
The entire V2 region of JRCSF is the primary determinant of JRCSF specificity towards PG9/PGT145 class of bNAbs. **a** Schematic representation showing swapping mutants of JRFL domains in JRCSF used in this experiment. **b** Sequence analysis of V2 domain of JRFL and JRCSF and position of mutants used in this experiment. **c** FACS-based cell surface staining assay of JRFL, JRCSF and the domain swapped mutants V1*C2 and V2*C2 with PG9 and PGT145 over a range of antibody concentrations. **d** Comparative binding of V2 domain mutants of JRCSF in a FACS-based cell surface assay with PG9 and PGT145 over a range of antibody concentrations. The *bar diagram* represents the binding of the respective antibodies to the wild type and different mutant Envs at 20 µg/ml of antibody concentration

In order to determine the minimal region in JRCSF, which imparts an advantage over JRFL in its ability to bind the PG9/PGT145 class of antibodies we used domain swapped mutants of JRCSF (Fig. 4a) in FACS based cell surface binding assays with PG9 and PGT145 (Fig. 4c). The mutant V1*C2 where the V1 and C2 domains of JRFL has been swapped into JRCSF binds efficiently to PG9 and PGT145 (Fig. 4c) while the mutant V2*C2 containing the V2 and C2 domains of JRFL swapped into JRCSF binds weakly to PG9 and PGT145 (Fig. 4c), suggesting that the V2 domain of JRCSF is critical for its ability to bind to this class of antibodies. We next determined the amino acid residues in the V2 domain of JRCSF that are critical for its ability to bind to this class of antibodies by replacing them with variants in JRFL. There are four amino acid changes between JRCSF and JRFL (K163T, N167D, K168E, and K191S) and one amino acid deletion (K187del) in the V2 domain (Fig. 4b). Two of these mutations viz N167D and K168E are in the strand C of V2 domain which plays a critical role in the ability of Envs to bind V1V2-directed bNAbs [39]. It should be noted here that Asparagines N156 and N160 are conserved between JRCSF and JRFL. The mutants were subjected to FACS based cell surface staining assay with PG9 and PGT145 antibodies (Fig. 4d). None of the mutants showed a defect in binding to PG9 and PGT145 as compared to JRCSF while JRFL showed weak binding as expected (Fig. 4d, left and right panel). JRCSF (K168E) was as efficient as JRCSF in its ability to bind PG9 and PGT145 (Fig. 4d). These results suggest that the entire V2 domain of JRCSF is required for binding to PG9/PGT145 class of antibodies.

### Restoration of CD4-bs-directed antibody binding activity in JRCSF

**Fig. 5:**
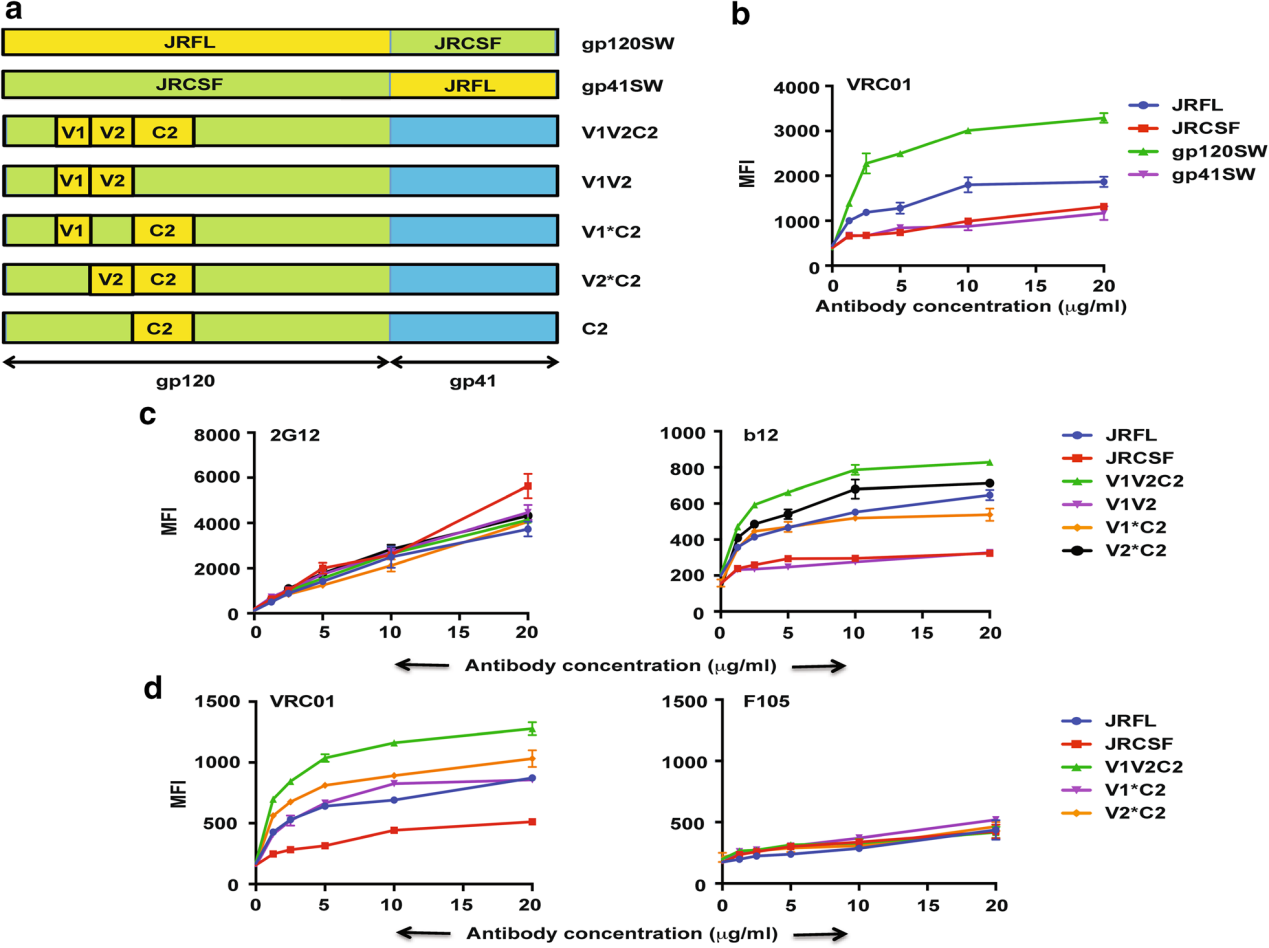
Domain swapping experiments to determine minimal region of JRFL, which confers CD4-bs-directed bNAb binding on JRCSF. **a** Schematic representation of domain swapped mutants. **b**–**d** FACS-based cell surface staining assay of JRFL, JRCSF and domain swapped mutants with the neutralizing Abs 2G12, VRC01, b12 and the non-neutralizing Ab F105 over a range of antibody concentrations

Cell surface expressed JRCSF binds weakly to CD4-bs-directed neutralizing antibodies (Fig. 1) as compared to JRFL. In order to use JRCSF for genetic vaccination and as a platform for immunogen design it is important that cell surface-expressed JRCSF binds efficiently to this category of neutralizing antibodies. It has been reported previously, using pseudovirus neutralization assay that swapping the C2 domain of JRCSF with that from JRFL results in efficient neutralization of JRCSF pseudoviruses by CD4-bs-directed antibodies [32]. Furthermore, Asn197 of JRCSF when mutated to Asp (N197D) restores ability of JRCSF to bind to CD4bs-directed antibodies in pseudovirus neutralization assay [32]. Asn197 may cause steric hindrance through the “umbrella effect” where a single glycan shields a local patch [40]. In order to investigate the minimal changes required to render JRCSF sensitive to CD4-bs-directed antibodies when expressed on the cell surface we generated a series of domain swapped mutants of JRCSF where different domains of JRFL were swapped into JRCSF as shown in Fig. 5a and tested these mutants in FACS based cell surface binding assays. We found that JRFL gp120 domain swapped into JRCSF (gp120SW) bound efficiently to the CD4-bs-directed antibody, VRC01 but not gp41SW (gp41 domain of JRFL swapped into JRCSF) (Fig. 5b). The efficiency of binding of JRCSF (gp120SW) to VRC01 was much higher as compared to even JRFL Env. We then tested the mutants V1V2C2 (V1, V2 and C2 domains of JRFL swapped into JRCSF), V1V2 (V1 and V2 domains of JRFL swapped into JRCSF), V1*C2 (V1 and C2 domains of JRFL swapped into JRCSF), and V2*C2 (V2 and C2 domains of JRFL swapped into JRCSF) along with JRFL and JRCSF for their ability to bind to 2G12 and b12 (Fig. 5c). All the wild-type and mutant Envs bound to 2G12 to a similar extent suggesting that the wild type and all Env variants were expressed on the cell surface to a similar extent (Fig. 5c, left panel). The mutants V1V2C2, V1*C2, V2*C2 but not V1V2 bound efficiently to b12 (Fig. 5c, right panel). The mutants V1V2C2 and V2*C2 showed higher binding to b12 than JRFL. We further tested the ability of these mutants along with wild-type Envs to bind to VRC01 and the non-neutralizing CD4-bs-directed antibody F105 (Fig. 5d). The mutants V1V2C2, V1*C2, V2*C2, bind efficiently to VRC01 but weakly to F105 (Fig. 5d). The binding of V1V2C2 and V2*C2 to VRC01 was found to be higher than that of JRFL. This suggests that the difference in binding to CD4-bs-directed bNAbs between JRFL and JRCSF is attributed primarily to the C2 domain of Env and is not due to a global disruption of the CD4-bs as there is no difference in binding to CD4-bs-directed non-NAbs like F105 between the wild-type and mutant Envs.

**Fig. 6:**
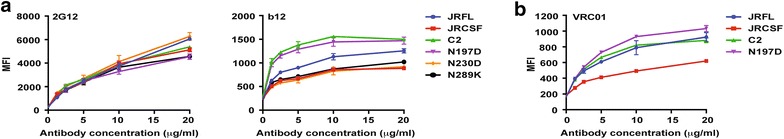
N197D mutation in JRCSF restores CD4-bs-directed bNAb binding to JRCSF. **a** FACS-based cell surface staining assay of JRFL, JRCSF and the mutants C2, N197D, N230D, N289K (all in JRCSF) with 2G12 and b12 over a range of antibody concentrations. **b** FACS-based cell surface assay of JRFL, JRCSF and the mutant JRCSF (N197D) with VRC01 over a range of antibody concentrations

In order to further demonstrate that the C2 domain of JRFL restored the ability of JRCSF to bind to CD4-bs-directed antibodies we tested the mutant C2 (JRFL C2 swapped into JRCSF) for its ability to bind b12 in the cell surface staining assay (Fig. 6a, right panel). We used binding to 2G12 as an expression control (Fig. 6a, left panel). The mutant C2 bound b12 better than JRFL itself (Fig. 6a, right panel) suggesting that swapping the C2 domain of JRFL into JRCSF restores the ability of this hybrid Env to bind to CD4-bs-directed bNAbs efficiently. We observed that the C2 region of JRCSF contains three N-linked glycosylation sites at position 197, 230 and 289 which are absent in JRFL. Furthermore, in pseudovirus neutralization assays it has been shown that mutation of Asn197 of JRCSF to Asp causes JRCSF (N197D) pseudotyped viruses to be efficiently neutralized by b12 and VRC01 [32] suggesting that glycosylation at Asn may play a critical role in the differential binding of JRFL and JRCSF to CD4-bs-directed bNAbs. Consequently, we mutated all three Asn residues at position 197, 230 and 289 to their counterparts in JRFL and tested these mutants for their ability to bind to b12 and 2G12 (Fig. 6a). The mutant JRCSF (N197D) is expressed to a similar extent as JRFL and JRCSF (Fig. 6, left panel) but binds more efficiently to b12 than JRFL (Fig. 6, right panel), comparable to the C2 mutant of JRCSF while the other two Asn mutants had no effect. Similarly, JRCSF (N197D) binds more efficiently to VRC01 than JRFL in cell surface binding assays (Fig. 6b). Taken together these studies demonstrate that mutating a single Asn at position 197 of JRCSF to Asp causes this mutant to bind to CD4-bs-directed bNAbs, b12 and VRC01 more efficiently than JRFL in cell surface staining assays and that this simple modification is sufficient to restore efficient binding of JRCSF to the CD4-bs targeted bNAbs. Asn197 in BG505 and X1193.c1 in their crystal structure with PGT122-35022 and VRC01 is glycosylated and can sterically inhibit by the “umbrella effect” [40].

### Restoration of CD4-bs in JRCSF does not disrupt overall conformation

**Fig. 7:**
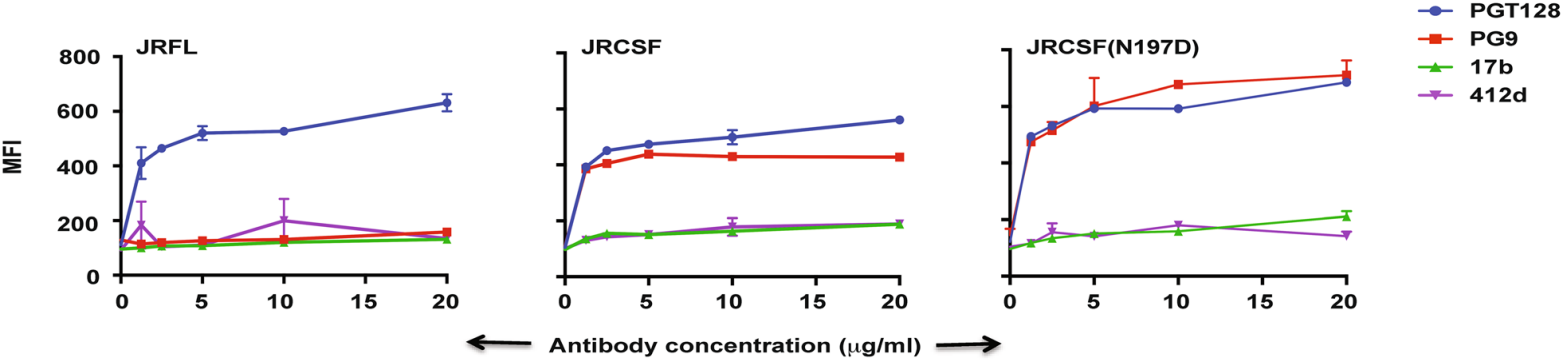
N197D mutant of JRCSF retains native conformation. FACS-based cell surface staining assay of JRFL, JRCSF and JRCSF (N197D) with glycan and quaternary epitope-dependent bNAbs PG9 and PGT128 and CD4i Abs 17b and 412d over a range of antibody concentrations

It has been previously reported that loss of the glycan at Asn197 of Env can affect its conformation [41] and allow CD4-independent entry and expose CD4-induced epitopes [42]. Here, we have shown that mutation of N197 in JRCSF to Asp not only restores binding to the CD4-bs antibodies but also increases its efficiency. We checked the binding to glycan and quaternary epitope-dependent bNAbs PG9 and PGT128 to N197D mutated JRCSF in comparison to JRFL and wild-type JRCSF (Fig. 7). In addition, we determined whether the CD4-induced epitopes get exposed in JRCSF (N197D) by studying its binding to the antibodies 17b and 412d in comparison to JRFL and wild-type JRCSF (Fig. 7). As shown in Fig. 7, JRCSF (N197D) binds efficiently to PG9 and PGT128 similar to wild-type JRCSF but poorly to the antibodies 17b and 412d. Taken together, these results suggest that JRCSF (N197D) maintains the native conformation.

### JRCSF is efficiently cleaved on the cell surface

**Fig. 8:**
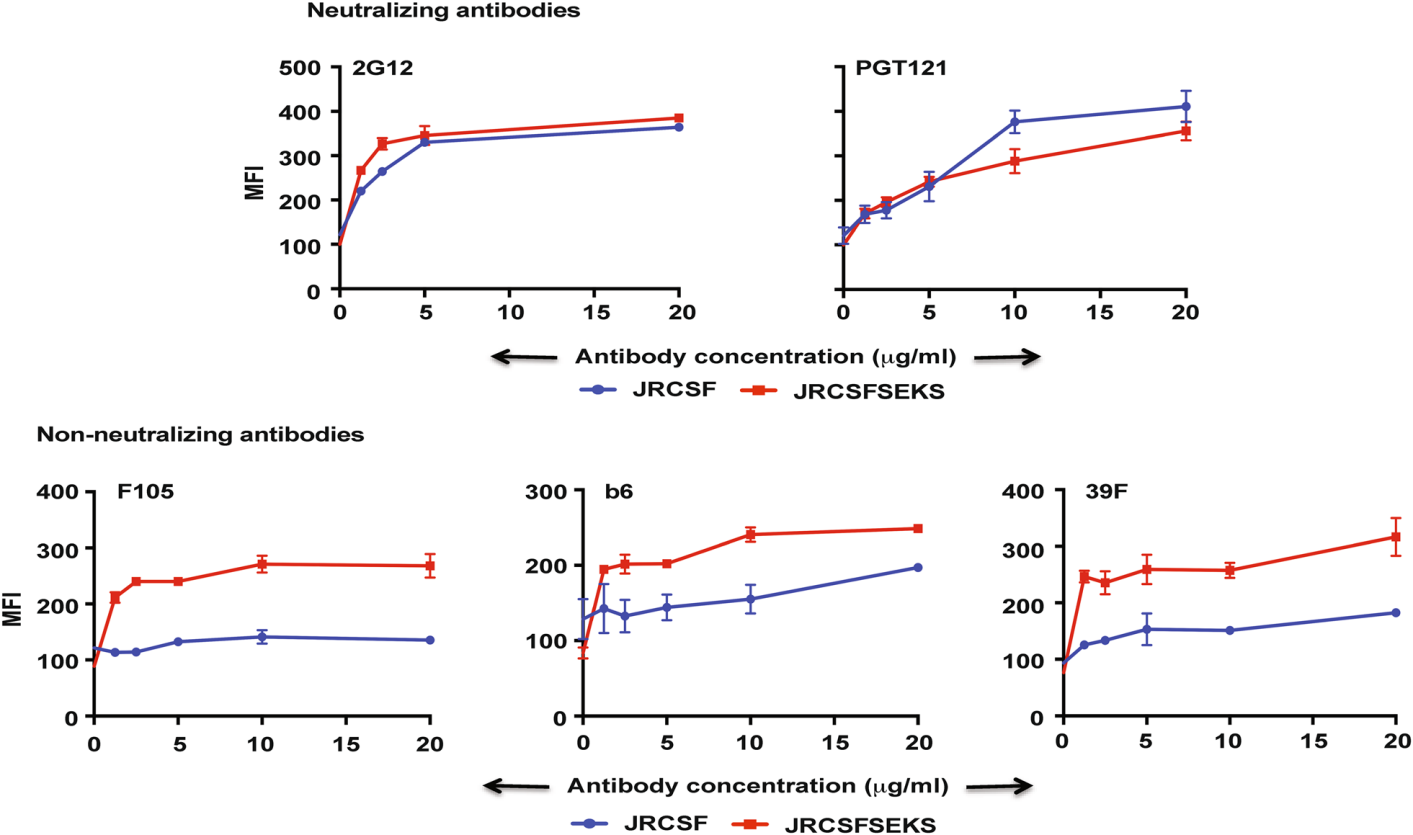
Mutation of REKR cleavage sequence to SEKS in JRCSF exposes non-neutralizing epitopes. Comparative binding of JRCSF and JRCSF (SEKS) to neutralizing Abs 2G12 and PGT121 and non-neutralizing Abs F105, b6 and 39F over a range of antibody concentrations by FACS-based cell surface staining assay

Naturally occurring, efficiently cleaved Envs like JRFL and 4-2.J41 when expressed on the cell surface bind preferentially to neutralizing antibodies over non-neutralizing antibodies [23, 24]. Since JRCSF appears to specifically display neutralizing epitopes, we, therefore, sought to find out whether like JRFL Env, JRCSF is efficiently cleaved on the cell surface. It has been demonstrated earlier that the conformation of efficiently cleaved Env is different from uncleaved Env as the latter binds to both neutralizing and non-neutralizing antibodies. We observed that JRCSF Env does not bind to non-neutralizing antibodies on cell surface and this is due to the efficient cleavage of JRCSF Env on cell surface. An alternative explanation could be that the epitopes for the non-neutralizing antibodies are not present in this Env. It has been previously demonstrated with other efficiently cleaved Envs, JRFL and 4-2.J41 that mutating the cleavage site, REKR of gp160 to SEKS results in cleavage-deficient Env and the uncleaved Env binds to non-neutralizing antibodies [23, 33]. In order to check for the presence of epitopes for non-neutralizing antibodies in JRCSF Env, we first mutated the cleavage site, REKR of JRCSF to SEKS and then used FACS-based cell surface staining assay to compare both wild type and the mutant Env for their ability to bind to non-neutralizing antibodies F105, b6 and 39F when expressed on the cell surface (Fig. 8). We also used 2G12 and PGT121 antibodies to determine the relative expression levels of JRCSF and JRCSF (SEKS) mutants as controls (Fig. 8). Both JRCSF and JRCSF (SEKS) mutant bound to 2G12 and PGT121 to a similar extent suggesting that they are expressed to a similar extent on the cell surface (Fig. 8, top panel). Furthermore, in contrast to wild type JRCSF, JRCSF (SEKS) mutant bound to the non-neutralizing antibodies F105, b6 and 39F with about twofold greater efficiency (Fig. 8, bottom panel) suggesting that epitopes for non-neutralizing antibodies in efficiently cleaved JRCSF are present and they are occluded in its cleaved form on cell surface. The epitopes become exposed as a result of conformational change only when the Env is rendered cleavage defective.

**Fig. 9:**
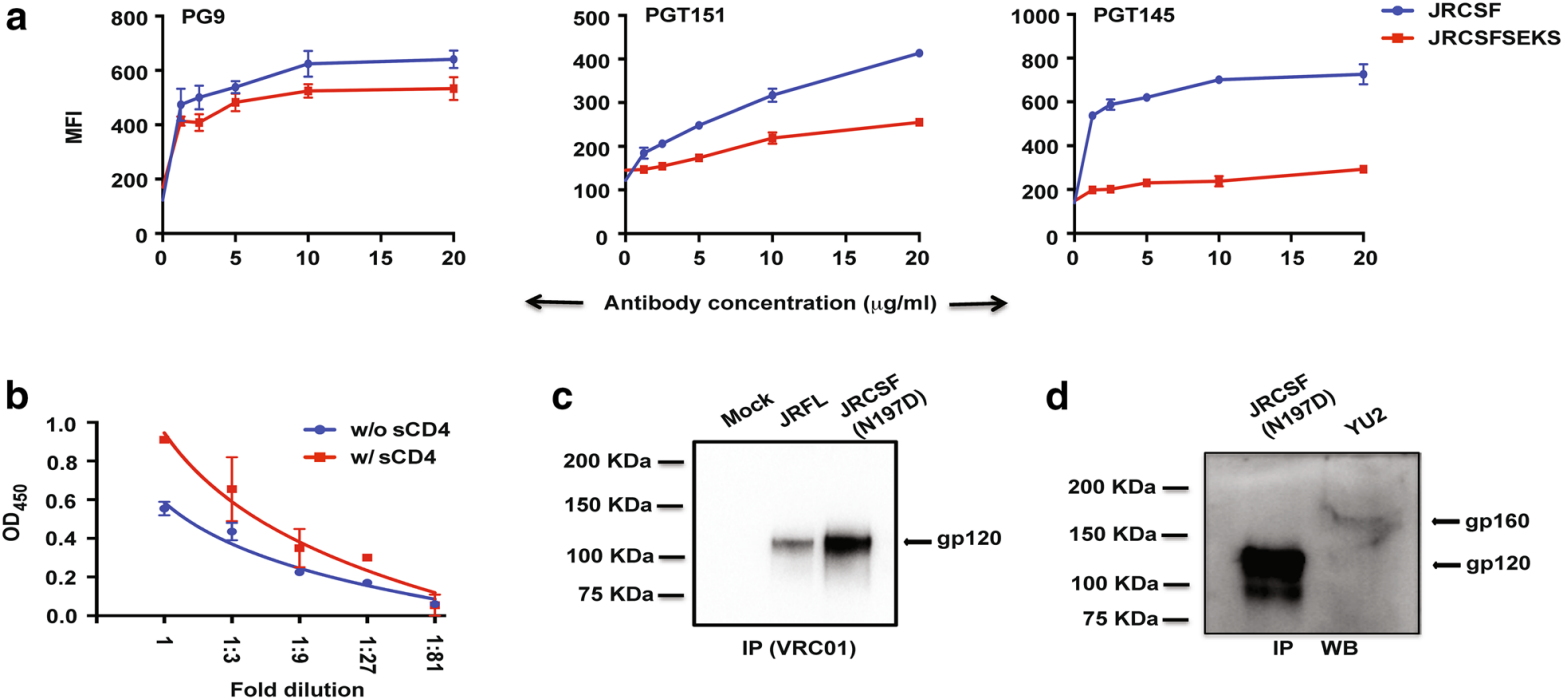
Cleavage property of JRCSF Env. **a** Cell surface staining assay of JRCSF and JRCSFSEKS with PG9, PGT151 and PGT145 over a range of antibody concentrations. **b** ELISA assay of gp120 shedding of JRCSF transfected 293T cells incubated with and without 50 μg/ml sCD4-183. **c** Western blot analysis of VRC01 immunoprecipitates of plasma membrane fraction of JRFL and JRCSF(N197D) transfected 293T cell lysates with anti-clade B Env rabbit Abs. **d** Western blot analysis of VRC01 immunoprecipitate of plasma membrane fraction of JRCSF(N197D) transfected 293T cell lysates and crude lysate of YU2 with anti-clade B Env rabbit Abs

We also tested JRCSF and JRCSF (SEKS) for their ability to bind to native, cleaved, trimer-targeted bNAbs PGT151 and PGT145. We have previously reported that JRFL (+) (cleavage-competent, tail-truncated JRFL) binds efficiently to PGT151 on the cell surface and this binding is considerably reduced upon mutating the gp160 cleavage site from REKR to SEKS [23]. As shown in Fig. 9a, both JRCSF and JRCSF (SEKS) bind to the glycan and quaternary epitope-dependent antibody, PG9 to a similar extent in cell surface antibody binding assay but JRCSF (SEKS) shows a twofold reduction in binding to PGT151 and a drastic reduction in binding to PGT145. This suggests that JRCSF, like JRFL, is present in its native, trimeric, cleaved conformation on the cell surface. In addition, we used sCD4-mediated gp120-shedding to demonstrate that JRCSF is efficiently cleaved on the cell surface. Cell surface expressed, efficiently cleaved Envs undergo conformational changes upon CD4 binding, which results in dissociation of the labile gp120-gp41 interaction and release of the gp120 moiety [23, 33]. Following expression of JRCSF on the cell surface of transfected cells, incubation with sCD4 was carried out and gp120 released into the supernatant in the absence and presence of sCD4 was measured by lectin mediated capture of Env followed by ELISA. Incubation with sCD4 results in about a twofold increase in the amount of gp120 shed (Fig. 9b) in the supernatant demonstrating that JRCSF is efficiently cleaved on the cell surface.

We then used immunoprecipitation (IP) assay, as described in materials and methods section, to determine biochemically whether JRCSF is efficiently cleaved on the cell surface. As we observed an extra non-specific protein band migrating close to the expected protein band of molecular weight of gp120 while performing the IP assay of wild type JRCSF Env with cleavage-independent, glycan-directed antibody, 2G12 (data not shown), we performed immunoprecipitation of antigenically more relevant Env, JRCSF (N197D) with cleavage-independent, CD4-bs-directed antibody, VRC01 for determining its efficiency of cleavage on cell surface. Plasma membrane (PM) fractions of mock-transfected, JRFL and JRCSF (N197D) Env transfected cells were immunoprecipitated with cleavage non-specific bNAb VRC01 as indicated (Fig. 9c) and bound proteins subjected to SDS page followed by western blot analysis. Both JRFL and JRCSF (N197D), immunoprecipitated with VRC01, shows a single band migrating between the 100 and 150 kDa markers (Fig. 9c). JRFL has been previously reported to be efficiently cleaved on the cell surface showing a single gp120 band [24] suggesting that this single band in the JRFL and JRCSF lanes constitute the gp120 subunit. To verify whether the single band represents gp120 from cleaved Env, we compared the immunoprecipitated PM fraction of JRCSF (N197D) to the crude cell lysate of the clade B env, YU2 which has been shown to be uncleaved previously [24]. YU2 shows a single slower migrating band representing gp160 suggesting that the band in JRCSF (N197D) immunoprecipitated lane is gp120 (Fig. 9d). YU2 expression is relatively low (see Additional file 1: Figure 2 for higher exposure). Taken together our data provide strong evidence that JRCSF is efficiently cleaved on the cell surface.

Immunization with soluble versions of Env proteins is a viable option to elicit neutralizing antibodies. Towards that goal we tested the SOSIP version of JRCSFN197D (JRCSFN197DSOSIP) in crude supernatant of transfected 293T cells (Additional file 2) for its ability to bind a panel of bNAbs (Additional file 1: Figure 3) by immunoprecipitation (IP). We observed that in the absence of furin, a majority of JRCSFN197DSOSIP is in the cleaved form (gp120). Co-transfection with furin and IP with VRC01 showed only gp120 band and co-migrated with bands obtained with other bNAbs, particularly the cleavage-specific, trimer selective bNAbs PGT145 and PGT151. We also obtained interaction with non-NAb F105, suggesting that like JRFLSOSIP [22], the JRCSFN197DSOSIP extract is a mixture of native-like Env trimers and non-trimers. Thus, JRCSFN197DSOSIP displays the epitopes of all classes of bNAbs tested (except VRC34 which does not bind efficiently in cell surfaces staining assay). We intend to purify native-like trimers of JRCSFN197DSOSIP from this heterogenous pool using positive (PGT145) or negative (F105) selection and analyze its properties by biochemical, EM and biophysical studies.

## Discussion

The currently available candidate vaccines generally lack the neutralization breadth [14, 15, 27, 43]. The diversity of HIV poses a major obstacle to developing a universally protective vaccine against HIV and prevention of AIDS. In order to contend with the diversity, there is a need to isolate cleaved, trimeric, Envs in appropriate conformation from multiple clades for designing of polyvalent vaccine candidates. However, the search for such Envs has been confounded by the rarity of Envs that are naturally and efficiently cleaved, and the difficulty of obtaining soluble, metastable versions of Envs that display all the requisite properties desirable in immunogen candidates. Efficient cleavage of the Env gp160 polypeptide negatively correlates with its binding efficiency to non-neutralizing antibodies but not to CD4-bs neutralizing antibodies. However, it positively correlates with glycan-directed conformation dependent broadly neutralizing antibodies and its ability to neutralize viruses pseudotyped with such Envs.

JRFL of clade B and the recently reported 4-2.J41 of clade C are the only known Envs, which are cleaved on the cell surface without requiring the co-expression of exogenous protease, furin. It is to be noted here that the efficiency of cleavage on cell surface may be screened by checking the binding ratio/stoichiometry between the CD4-bs directed neutralizing antibodies, b12 and/or VRC01 versus CD4-bs directed non-neutralizing antibodies, F105 and/or b6 [23, 24]. Surprisingly, JRCSF Env does not bind efficiently to CD4-bs-directed antibodies yet binds efficiently to cleavage dependent antibody, PGT151 suggesting that JRCSF does not exhibit the associative property of binding to CD4-bs antibodies and cleavage. Furthermore, the cleavage efficiency was also proved by CD4-induced shedding and other biochemical data. Therefore, to broaden the repertoire of available Env immunogen candidates, it is desirable that more Envs with similar properties are identified.

In this study we show for the first time, that JRCSF is efficiently cleaved on the cell surface and that this property of JRCSF correlates with its property to preferentially bind bNAbs when expressed on the cell surface, which is in agreement with previous reports of neutralization capacity of JRCSF pseudotyped viruses by bNAbs [34]. The fact that JRCSF presents itself on the cell surface in the correct, trimeric, native conformation makes it an ideal starting point to design immunogens aimed at eliciting neutralizing antibodies and also to design soluble variants of this Env similar to JRFL [22] and 4-2.J41 [23]. Also, JRCSF may be more “native-like” than JRFL in that it efficiently binds to PG9/PGT145 class of bNAbs even better than JRFL (E168K). The diversity of the variable regions 1 and 2 (V1/V2) of Env plays an important role to evade neutralizing antibody response. However, structural evidences have shown that N-linked glycosylation either clustered or dispersed along with strand-connecting loops are involved in recognition of glycan-directed conformational antibodies [39, 40, 44] Although, N156 and N160 in JRFL and JRCSF are conserved, there are some differences in strand C between these two Envs (Fig. 4b). However, single amino acid mutations in V2 (N167D and K168E) did not abolish binding of JRCSF to V1V2 bNAbs. We do not rule out the possibility that the double mutant or other differences in the residues in the epitope for this class of bNAbs will have an effect on binding.

Furthermore, JRCSF, which binds weakly to CD4-bs-directed antibodies, can be made to bind this class of bNAbs efficiently by incorporating the N197D mutation without disrupting its native conformation. It is to be noted here that the efficiency with which mutant JRCSF binds CD4-bs bNAbs is more than that of JRFL. Taken together our findings suggest that JRCSF can be added to the pool of efficiently cleaved, naturally occurring Envs that could be used to elicit neutralizing antibodies. The C2 dependent binding of CD4-bs-directed antibodies to JRCSF has been shown in virus context [32]. However, in the context of designing immunogens, it is important to determine whether JRCSF displays its properties when expressed either on cell surface or in its soluble form. Moreover, for the first time, we show the V2 dependent binding of broadly neutralizing antibodies to JRCSF on cell surface. In this study, we also demonstrate that JRCSF displays epitopes for newly isolated and relatively abundant broadly neutralizing antibodies (except VRC34) that have been identified from HIV-1 infected individuals. Furthermore, its binding ability to CD4-bs antibodies can be enhanced by a simple modification without altering its binding ability to broadly neutralizing antibodies mentioned above.

Significant progress has also been made in generating soluble versions of Env proteins. Modified versions of JRFL, BG505, 16055 (clade C) and B41 (clade B) have been used to obtain metastable, purified proteins that display properties of improved immunogen candidates. We conclude that efficiently cleaved modified JRCSF Env would be a good starting point for designing an immunogen with the goal of eliciting bNAbs, both as membrane bound form for the purpose of genetic vaccination as well as in its soluble form for protein boost, as it specifically displays nearly all the epitopes that bind to broadly neutralizing antibodies. In this direction, we have generated JRCSFN197DSOSIP and shown that the crude soluble protein binds to a majority of tested bNAbs. In future, we intend to purify this protein and subject it to various biochemical, EM and biophysical assays in order to investigate its properties that are suitable for immunization studies in animal models.

## Conclusion

Viral diversity is a significant impediment in developing a universal vaccine against HIV-1. It is likely that such a polyvalent vaccine will require that immunogens based on the viral glycoprotein Env be developed from a wide variety of HIV-1 sub-types. However, currently available candidate immunogens have limited neutralization breadth and potency necessitating the search for newer Envs with desirable antigenic properties. Env-based immunogens, suitable for vaccination, can be delivered both by DNA-based methods as well as soluble proteins. Here, we report the membrane bound properties of a clade B Env, JRCSF that is suitable for immunogen design. Cell surface expressed efficiently cleaved Envs are the closest mimic of native, trimeric forms of Env and co-relate with their ability to preferentially expose only broadly neutralizing epitopes. Previously, only two Envs, JRFL (clade B) and 4-2.J41 (clade C) were reported to be efficiently cleaved on the cell membrane. In this report, we show that JRCSF, another clade B Env, is efficiently cleaved on the cell membrane and displays greater variety of broadly neutralizing epitopes as compared to JRFL, sometimes with superior binding efficiency and including quaternary and glycan-dependent epitopes. Our findings present evidences that JRCSF Env can be utilized as a suitable reagent for genetic vaccination as well as for designing soluble immunogens based on its efficient cleavage and superior antigenic profiles upon modification.

## Additional files

**Additional file 1: Figure 1.** Amino acid sequence comparison of JRFL and JRCSF full length polypeptide. Amino acid differences marked by *.** Figure 2.** Higher exposure of western blot analysis of VRC01 immunoprecipitate of plasma membrane fraction of JRCSF (N197D) transfected 293T cell lysates and crude lysate of YU2 with anti-clade B Env rabbit Abs. **Figure 3.** Soluble JRCSFN197DSOSIP displays different classes of bNAb epitopes. JRCSFN197DSOSIP-transfected cell supernatant subjected to immunoprecipitation with different bNAbs and F105 were analyzed by western blot analysis using rabbit anti-clade B Env antibodies as probes. Supernatant from furin co-transfected cells shown.

**Additional file 2.** Materials and methods.

## Abbreviations

HIV-1: human immunodeficiency virus type I
Env: envelope
bNAb: broadly neutralizing antibody
non-NAb: non-neutralizing antibody
FACS: fluorescence activated cell sorting
PM: plasma membrane
IP: immunoprecipitation
CD4i: CD4 induced
