# Supplementary material for: Membrane bound modified form of clade B Env, JRCSF is suitable for immunogen design as it is efficiently cleaved and displays all the broadly neutralizing epitopes including V2 and C2 domain-dependent conformational epitopes
Source: Retrovirology. 2016 Nov 21;13:81. doi: 10.1186/s12977-016-0312-7 (PMC5117575; doi:10.1186/s12977-016-0312-7)
Supplement: Supplementary file 2 — Additional file 2. Materials and methods. [file 12977_2016_312_MOESM2_ESM.rtf]

Soluble protein immunoprecipitation
	293T cells were transfected with plasmids pSVIII-JRCSFN197DSOSIP and pc-tat with or without plasmid expressing furin. 36-48 hours post-transfection, supernatant was harvested, filtered through 0.22 micron filter and about 1 ml each was subjected to immunoprecipitation with different neutralizing and non-neutralizing antibodies O/N with rotation at 4 0C. Antigen-antibody complexes were captured with Protein G resin (G Biosciences) for 1 hour with rotation at 4 0C, washed with PBS + 1% Triton-X and subjected to western blot analysis with rabbit anti-clade B Env antibodies (ABLinc) as probes.
